# The Antimicrobial Effect of Pomegranate Peel Extract versus Chlorhexidine in High Caries Risk Individuals Using Quantitative Real-Time Polymerase Chain Reaction: A Randomized Triple-Blind Controlled Clinical Trial

**DOI:** 10.1155/2021/5563945

**Published:** 2021-08-30

**Authors:** Benoy Jacob, Nivedhitha Malli Sureshbabu, Manish Ranjan, Aishwarya Ranganath, Riluwan Siddique

**Affiliations:** Department of Conservative Dentistry and Endodontics, Saveetha Institute of Medical and Technical Sciences, Velappanchavady, Poonamalle High Road, Chennai, Tamil Nadu 600077, India

## Abstract

The aim of the present study was to compare the antibacterial effectiveness of chlorhexidine and PPE oral rinse on *S. mutans*, *Lactobacilli*, and *Veillonella*, in clinical salivary samples of patients with advanced stages of dental caries at baseline and two and four weeks with PCR technique. This triple-blind randomized clinical trial involved 60 high caries risk adult patients, 19–59 years of age, randomly allocated into two groups of 30 subjects each. The intervention group received pomegranate peel extract mouthwash, whereas the control group received chlorhexidine mouthwash. Unstimulated pooled saliva was collected from the floor of the mouth before and after the intervention. The quantitative real-time polymerase chain reaction was employed to analyze the bacterial copies of each salivary sample at baseline and two and four weeks. The significance level was fixed at 5% (*α* = 0.05). Overall comparison of antimicrobial effectiveness across both groups revealed insignificant outcomes. The control group evinced a significant reduction in *S. mutans* between a specific time, i.e., baseline and 4 weeks (*p*=0.043). PPE oral rinse as a natural product or ecological alternative was effective in disrupting activity across all microorganisms tested in this triple-blind RCT; however, the nutraceutical, when compared to chlorhexidine, was not as effective against *S. mutans*.

## 1. Introduction

For decades, the use of chlorhexidine (CHX) and fluorides in prevention and treatment strategies has been directed at caries control and progression [1]. Having said that, barring few ineludible side effects pertaining to tooth staining, burning sensation, etc., long-term implementation of the broad spectrum antimicrobial (CHX) incites the risk of microbial resistance, and corroboration and skepticism pointing toward depletion of entire microflora endangering commensal populace also loom large [2, 3]. An ecological approach wherein efforts equipoise the symbiotic aura in the oral microbiome has been preferred over prevalent ones so as to maintain complete tranquility among the colossal mélange of oral microbiota. [4] A noninvasive ecological stratagem, propelling natural products into the cariological verse in a bid to deliver potentially active cariostatic catalytic agents in the form of mouthwashes, gels, varnishes, or chewing gums have been adopted, researched, and enforced in order to proselytize cariogenic microbial demise [5, 6]. Of all known natural products researched to date, the pomegranate, which possesses nutritional and medicinal health benefits galore, has the capacity to vehemently respond to a plethora of human oral and/or systemic illnesses [7–9].

In the literature, although in vitro experiments are plenteous, in vivo studies associating the comparison of the antibacterial efficacy of either Pomegranate Peel Extract (PPE) or other natural product oral rinses and CHX with *S. mutans* and/or other cariogenic strains are few and far between. On another note, caries-related studies involving the adult population as subjects are relatively exiguous as well [10]. However, in existing in vivo studies, according to the respective authors, there persisted few limitations; also, much credence has been given to *S. mutans* alone although the carious disease process is generally an outcome of capricious interactions among different types of bacteria. All things are considered; the aim of the present study was to compare the antibacterial effectiveness of chlorhexidine and PPE oral rinse on *S. mutans*, *Lactobacilli*, and *Veillonella* in clinical salivary samples of patients with advanced stages of dental caries at baseline (T_0_), two weeks (T_1_), and four weeks (T_2_) by employing a molecular technique, the quantitative real-time polymerase chain reaction (qPCR). A molecular approach (qPCR) was employed in this study on account of persistent reports of high sensitivity and specificity in the detection and quantification of microorganisms [11, 12].

## 2. Methods

### 2.1. Study Design

**Figure 1:**
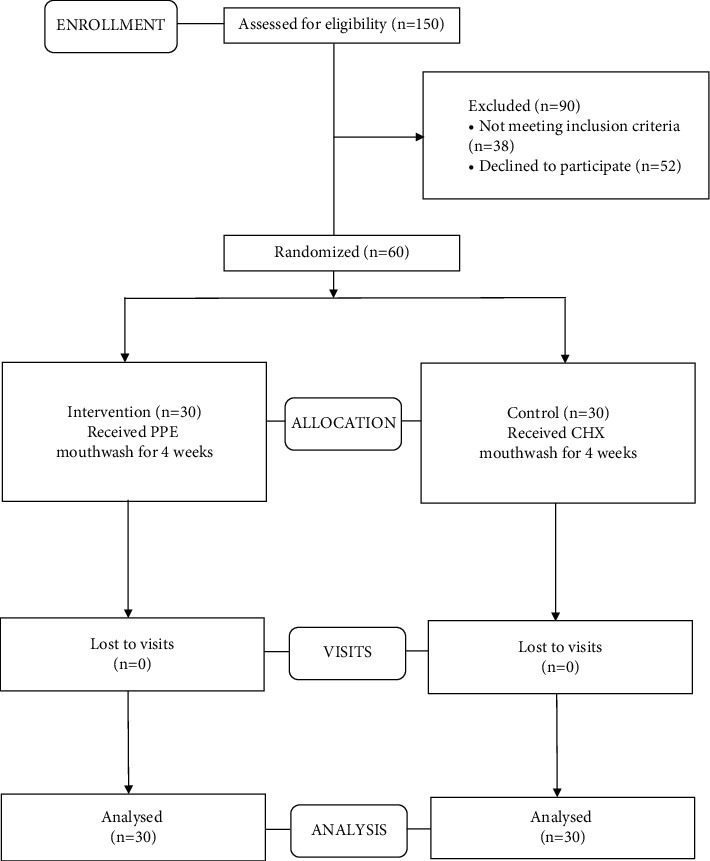
CONSORT flow chart.

The present study was performed as a triple-blind randomized clinical trial (RCT) wherein the primary investigator (clinician), participants, and the data analyzer were oblivious to the treatment or intervention (type of oral rinse) being rendered. Sample collection was performed by a single well-trained operator (clinician) throughout the completion of the study. This clinical trial conformed to the guidelines of the revised Consolidated Standards of Reporting Trials (CONSORT) statement [13]. CONSORT flow diagram is represented in Figure 1.

### 2.2. Setting and Location

Following the screening of 150 patients (walk-in), a total of 60 subjects who fulfilled the inclusion criteria (described later) were selected from the Department of Conservative Dentistry and Endodontics, Saveetha Dental College and Hospitals, Saveetha Institute of Medical and Technical Sciences (SIMATS), Chennai, Tamil Nadu, India.

### 2.3. Ethical Considerations

This triple-blind RCT conformed to the ethical guidelines of the Declaration of Helsinki (1975) and was approved by the Ethics Committee of the Saveetha Institute of Medical and Technical Sciences, Chennai (Ethical code: SRB/SDMDS16ODS/14, Clinical Trials Registry- India (CTRI), registration number: CTRI/2019/04/018387). All subjects agreed to participate and signed the informed consent form.

### 2.4. Sample Size

The sample size calculation was done based on erstwhile data procured from a former study which compared reduction in *S. mutans* count following the administration of control (CHX) and intervention (PPE), respectively [14]. For an *α* value equal to 0.05 and a power of 80% (*β* = 0.2), *z*_1_ − (*α*/2) = 1.96, and *z*_1_ −  *β*=0.84, the total sample size of 52 was standardized to 60 and distributed into 2 groups of 30 subjects each.

### 2.5. Inclusion and Exclusion Criteria

Inclusion criteria were as follows: 19- to 59-year-old high caries risk adult patients with DMFT (D = decay, M = missing, FT = filled teeth) > 6. To categorize the subjects as “high caries group,” dental examination was performed to detect DMFT; additionally, the American Dental Association's (ADA) Caries Risk Assessment (CRA) form was also utilized with great effect to rationalize the selection process. The ADA CRA form aided in recognizing and recording each candidate's fervent affinity toward sugary food and drink besides the daily recommended fluoride exposure.

#### 2.5.1. Exclusion criteria

Those already on a mouthwash regimen or consuming any drug that reduces salivary flow, medically compromised individuals, and patients undergoing orthodontic therapy or dental rehabilitation were excluded from the study. The exclusion criteria also laid emphasis on patients under any antibiotic or anti-inflammatory drugs during the past 1 month, alcoholics, smokers, paan, or gutka chewers (former and current). Subjects who portray missing teeth for reasons other than dental caries, undergone radiation therapy, or/and present with salivary gland-related disorders were also excluded from the study.

### 2.6. Randomization, Allocation Concealment, and Blinding

The included subjects were randomly allocated by block randomization, which was done well in advance using a table of random numbers by a third party with block sizes being unknown to the investigators until the completion of the study. SNOSE (Sequentially Numbered Opaque Sealed Envelopes) method for allocation concealment was implemented, ensuring complete anonymity of the respective groups [15]. Compact cards with randomized group codes (A or B representing CHX or PPE, known only to the third party) were retained in dark-colored sequentially numbered sealed envelopes. The third party, who was not related to the study, was aware of the coding. Furthermore, it is to be also noted that the third party was assigned to dispense both PPE and CHX mouthwashes into 300 ml bottles and code them into A or B as well. At the commencement of the experiment, the sealed envelope was opened by the clinician. Upon unsealing, corresponding to the card obtained, a coded (A or B) amber-colored 300 ml bottle containing either of the interventions, i.e., CHX (Hexidine 0.2%) or PPE, both arranged by the third party, was numbered according to the number on the envelope and given to the subjects. Neither the clinician nor the subjects were aware of the type of mouthwash being administered. Each subject was asked not to abstain from their normal diet, and most importantly, routine oral hygiene measures were to be continued as well, uninterrupted. At the culmination of the triple-blind RCT and analysis of samples by the data analyzer (also blinded), the third party unveiled the coded bottles for interpretation of results.

### 2.7. PPE Oral Rinse Preparation

The pomegranate fruit peel extract and mouthwash were prepared at the Department of Biochemistry, SIMATS, Chennai. The pomegranate cultivar used in the present study was particularly selected as numerous studies have time and again proclaimed its antioxidant and antibacterial potential [16–18]. Pomegranate fruits of Ganesh variety were procured from the local market of Chennai. The methodology used in this study was equivalent to the one we followed in our in vitro study (yet to be published). The fruits were washed thoroughly, and peels were peeled off manually, cut into small pieces, and dried in a hot air oven at 40°C for 48 hours. The dried peels were then ground in an electric grinder for 30 seconds to fine powder form (40-mesh size). Hydroethanolic (HE) (70% ethanol and 30% water) PPE was prepared to employ the Soxhlet extraction method [19], wherein 200 g of pomegranate peel powder and 450 ml of solvent (315 ml ethanol; 135 ml distilled water) were used to formulate the concoction. A minimum temperature range and time period capable of obtaining maximum yield (phenolics, flavonoids, polyphenols, etc.) from the HE extract, in line with previous studies concerning the extraction of pomegranate peel and other natural products, were maintained throughout the extraction process [16, 17, 20]. The solvent mixture was heated at 30°–50°C for 4 hours, following which the extract was filtered through Whatman No. 41 filter paper. After filtration, the extract was concentrated under vacuum at 40°C using a rotary evaporator [17], keeping in mind the 20/40/60 rule, so as to ensure quick and complete evacuation of solvent under controlled conditions while holding the essential compounds intact [21]. The 10 g of extract thus obtained was then dried and centrifuged at 3500 rpm for 8 minutes. Prior to the preparation of mouthwash, an in vitro study (yet to be published) was done at first to evaluate the antimicrobial efficacy of the HE PPE against *Streptococcus mutans* and *Lactobacillus acidophilus*, the results of which demonstrated well-defined zones of inhibition, when compared to CHX. In the preliminary study, we determined the Minimum Inhibitory Concentration (MIC) and Minimum Bactericidal Concentration (MBC) of PPE against both bacterial strains using the microbroth dilution method. The MBC for *S. mutans* was shown to be 5 mg/ml and that of *L. acidophilus*, 10 mg/ml. For the triple-blind RCT, the entire process was repeated with fresh pomegranate fruits until enough quantity was obtained for the preparation of mouthwash. Then, the extract was prepared in greater quantities, dispensed in sterile Petri dishes, sealed, and stored in a freezer at −20°C until further use. For formulating the PPE mouthwash, 30 grams of PPE was dissolved in 300 ml of distilled water along with 100 mg of sodium saccharin (sweetener), 50 mg of crushed menthol crystals (flavor), 100 mg of sodium benzoate (preservative), and 3 ml of glycerin (humectant) [22]. Solvents/chemicals used were of analytical grade, procured from Merck, Mumbai, India. All contents in the mixture were thoroughly stirred, and the final solution was sieved into sterile 300 ml amber-colored bottles to be handed over to the candidates (described later).

### 2.8. Saliva Collection

A stringent protocol was followed for saliva collection, sampling, and transport. Sample collection was performed by a single well-trained clinician throughout the completion of the study. Preliminary to the onset of the study, informed consent was obtained, and subjects were educated about the importance of each aspect of the study and how their coopetition would positively impact its outcome. On that note, all subjects were instructed well in advance to refrain from all modes of drinks and foodstuffs 1 hour prior to saliva collection. Saliva collection, storage, and transport for processing were done by the primary investigator. Each candidate was seated on the dental chair, relaxed and still, following which they were to rinse their mouth well with distilled water for 1 minute and expectorate. The time of saliva collection was austerely maintained between 8 and 11 am. Unstimulated pooled saliva (2 ml) was aspirated manually using sterile Dispovan syringes, transferred into microcentrifuge tubes, placed in the tube rack, and immediately kept inside a portable refrigerator with ice packs for transport to the laboratory for processing. Microcentrifugation tubes used to store saliva samples were numerically marked with a black marker pen according to the participant number (same number as envelope and bottle, as described earlier), and all tubes were marked with the equivalent number as well as a succeeding alphabet depicting each visit. For example, suppose that if the baseline saliva sample collected from a subject is marked as 1, the corresponding samples following further visits for the same subject, i.e., 1^st^ and 2^nd^ visit samples, would be marked as 1a and 1b.

Following prerinse saliva sample collection, upon unsealing of concealed envelopes (as described earlier), subjects were given the respective coded amber-colored 300 ml bottles of oral rinse and instructed to dispense 10 ml of the solution in a measuring cup which was provided to them, swish around the mouth for 30 seconds at night before bedtime, and expectorate. This was to be repeated for the next 4 weeks. Periodic telephonic reminders (calls and text messages) were meted out to each candidate with respect to mouthwash usage. The 1^st^ visit was scheduled at the end of the second week during which the 1^st^ postrinse saliva samples were taken. The second visit was programmed 2 weeks after the 1^st^ visit, i.e., at the end of 4 weeks. During the second visit, i.e., at the end of the 4^th^ week, the second (final) postrinse unstimulated saliva samples were collected. On completion of the clinical trial, subjects were solicited for feedback concerning the corresponding oral rinses given to them. Each pragmatic response was recorded. For ease of sample identification in this article, samples collected at baseline, two weeks, and four weeks were designated as T_0_, T_1_, and T_2_, respectively.

### 2.9. qPCR

#### 2.9.1. DNA Extraction

Saliva samples were collected in a 1.5 ml sterile DNase/RNase free tube and stored at 4°C until transported to the laboratory for DNA extraction. At the time of DNA extraction, 200 *μ*l of saliva was centrifuged at 10,000 rpm for 3 minutes at room temperature. The pellet thus obtained was washed once with sterile 1X PBS (Phosphate Buffer Saline, pH7.5) (Cat#P3813, Sigma-Aldrich, USA) before being subjected to DNA extraction with lysis buffer containing 100 mM of Tris (pH8), 25 mM EDTA, and 2% SDS digested with 10 mg/ml of lysozyme (Cat#L6876, Sigma-Aldrich, USA) at 37°C for 30 minutes. Following cell lysis, 20 mg/ml of Proteinase K was added, and the lysates were incubated at 57°C for 2 hours to digest all protein components present in the lysate. Subsequently, the lysates were transferred to DNA extraction columns as per the recommendation of the manufacturer after the addition of binding buffer (Cat# NA2110, Sigma-Aldrich, USA). The total amount of DNA present in each of the samples was quantified with a Qubit fluorometer (Life Technologies, USA) [23].

#### 2.9.2. Amplification and Quantitation of *S. mutans*, *Veillonella*, and *Lactobacillus*

In order to identify the quantitative presence of the above bacteria in the saliva samples, an equal concentration (1 nanogram) of total genomic DNA was subjected to real-time polymerase chain reaction (PCR) amplification with a pair of genus-specific primers as shown in Table 1 [24–26], where the sets of primers that are present within the 16S rRNA gene were used for each of the bacteria.

10 *μ*M of each of the primers was added to BRYT green RT-Master Mix (Cat# A6001, Promega, Madison, WI, USA) in 20 *μ*l reaction, and samples were analyzed in rotor gene Q real-time PCR equipment (Qiagen, Germany). The following universal amplification condition was used: after an initial denaturation at 95°C for 10 min, samples were amplified for 40 cycles at 94°C for 20 s, 56°C for 20 s, and 72°C for 20 s.

*(1) Establishment of Standard*. In order to quantitatively determine the copy numbers of each bacterium (relative to each other and among the samples), a standard curve was established with serial dilutions of PCR product amplified from V5-V6 region of 16s rRNA gene representing 789 to 1068 base pairs of *E. coli* genome. The following pair of primers was used: forward: TAGATACCCSSGTAGTCC (789–806), reverse: CTGACGRCRGCCATGC (1053–1068). The amplification produces a 279 base pair PCR product. The following amplification condition was used: after an initial denaturation at 95°C for 10 min, samples were amplified for 35 cycles at 95°C for 30 seconds, 55°C for 30 seconds, and 72°C for 30 seconds with a final extension at 72°C for 4 minutes. The V5-V6 PCR amplicon was gel purified (cat#NA1111, Sigma-Aldrich, USA) and eluted in 40 *μ*l of elution buffer. The concentration of gel eluate was determined by quantifying 1 *μ*l of the eluate by Qubit fluorometer (Invitrogen, Austria) using QuantiFluor ONE dsDNA system (cat#E4871, Promega, USA). The copy number of PCR amplicons present in nanograms of V5-V6 gel eluate was determined by using the following formula:

(1) $$\begin{array}{c} \frac{\left(\text{nanograms per microliter}\right)\times 6\text{.}022\times 10^{23}}{\left(\text{length of amplicon in base pairs}\right)\times 1\times 10^{9}\times 650}\text{.} \end{array}$$

After determining the copy numbers, serial dilutions of the V5-V6 eluate were made to obtain concentrations from 1 × 10^6^ to 1 × 10^1^. These serially diluted samples were then analyzed by real-time PCR in the presence of QuantiNova SYBR Green PCR Kit (Cat#208052, Qiagen, Germany) in a Qiagen 5-plex rotor gene real-time PCR system to establish a linear standard graph. The following amplification condition was used: after an initial denaturation at 95°C for 5 minutes, the standards were subjected to 40 cycles of amplification at 95°C for 15 seconds and 60°C for 30 seconds. The linear standard curve thus obtained was stored in the system to be used as a reference during the sample amplification process. Saliva samples collected from patients were processed for DNA extraction as described in the methods section. DNA thus extracted was quantified with a Qubit fluorometer to determine the concentration of DNA in each sample. The DNA concentration among the samples was found to vary and was in the range of 0.1 nanograms per microliter to 9 nanograms per microliter. Such variation is indeed expected as the amount of DNA obtained in each sample depends on the number of cells present in the saliva sample. All samples, regardless of the concentration, were subjected to amplification as the PCR technique is sensitive enough to amplify even from picogram levels of DNA.

**Figure 2:**
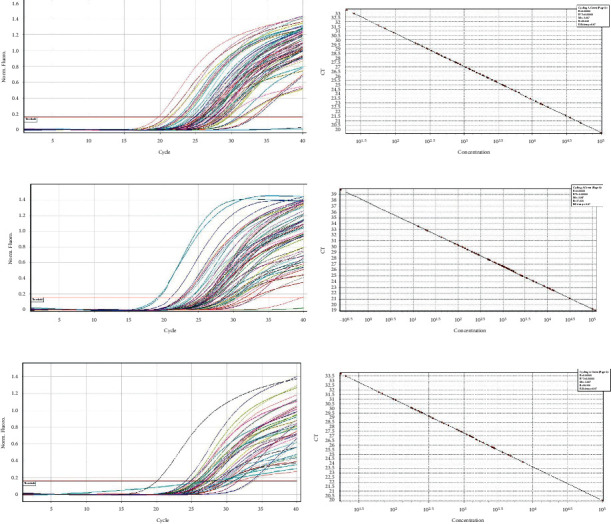
(a) *S. mutans* samples 1–23 amplification curve. (b) *S. mutans* samples 1–23 standard slope. (c) *S. mutans* samples 24–46 amplification curve. (d) *S. mutans* samples 24–46 standard slope. (e) *S. mutans* samples 47–60 amplification curve. (f) *S. mutans* samples 47–60 standard slope.

**Figure 3:**
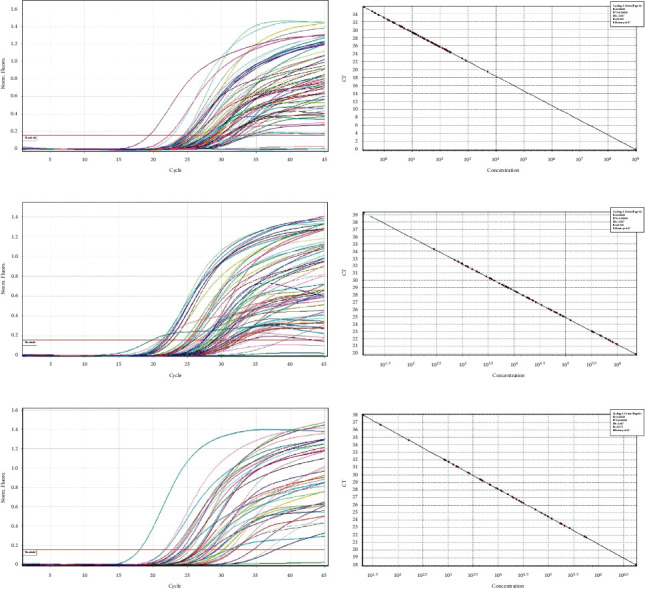
(a) *Lactobacillus* samples 1–23 amplification curve. (b) *Lactobacillus* samples 1–23 standard slope. (c) *Lactobacillus* samples 24–46 amplification curve. (d) *Lactobacillus* samples 24–46 standard slope. (e) *Lactobacillus* samples 47–60 amplification curve. (f) *Lactobacillus* samples 47–60 standard slope.

**Figure 4:**
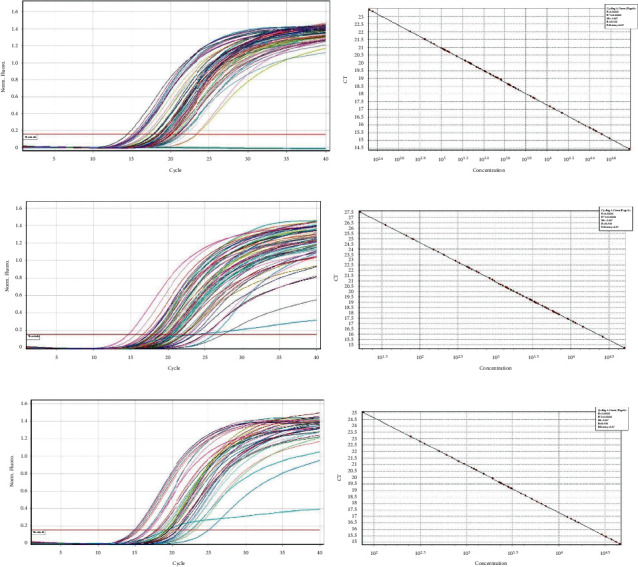
(a) *Veillonella* samples 1–23 amplification curve. (b) *Veillonella* samples 1–23 standard slope. (c) *Veillonella* samples 24–46 amplification curve. (d) *Veillonella* samples 24–46 standard slope. (e) *Veillonella* samples 47–60 amplification curve. (f) *Veillonella* samples 47–60 standard slope.

*(2) Quantitative PCR to Determine the Presence of Bacteria in Saliva Samples*. 1 *μ*l to 3 *μ*l of the DNA sample at a working concentration of 0.6 nanograms was used as template in a 20 *μ*l reaction volume in the presence of BRYT green-based fluorescence detection protocol in Qiagen RotorGene Q real-time PCR system. Amplification was performed as described in the methods section. The quantitative presence of each bacterium among the samples was determined by comparing the normalized fluorescence value with that of a linear standard graph obtained from running known concentration of control DNA (Figures 2(a)–2(f), 3(a)–3(f), and 4(a)–4(f)). The entire comparison procedure was performed with the in-built Qiagen RotorGene Q real-time PCR system software. Upon comparison, the software expresses the quantity of each bacterium as copy numbers. For example, if sample “A” has a higher concentration of *Lactobacillus* relative to sample “B,” sample “A” will produce higher fluorescence than sample “B.” The software detects this higher fluorescence in sample “A” and expresses the same as higher copy number of *S. mutans* in sample “A.”

*(3) Data Analysis*. The copy numbers of bacteria thus obtained from quantitative runs were analyzed to understand the relative presence of each bacterium among the two groups of samples.

### 2.10. Statistical Analysis

The normality tests Kolmogorov–Smirnov and Shapiro–Wilk revealed that all variables except age did not follow the normal distribution. Therefore, to analyze the data, both parametric and nonparametric methods were applied. For variables that did not follow the normal distribution, and to compare between groups, the Mann–Whitney test was applied. To compare values between all three time points, the Friedman test for repeated measures was used. Bonferroni adjusted *p* values were calculated for pairwise comparisons between two time points. To compare mean age between groups, an independent sample *t*-test was used. To compare proportions between groups, a Chi-square test was applied. To analyze the data, SPSS (IBM SPSS Statistics for Windows, Version 23.0, Armonk, NY: IBM Corp. Released 2015) was used. The significance level was fixed at 5% (*α* = 0.05).

## 3. Results

**Figure 5:**
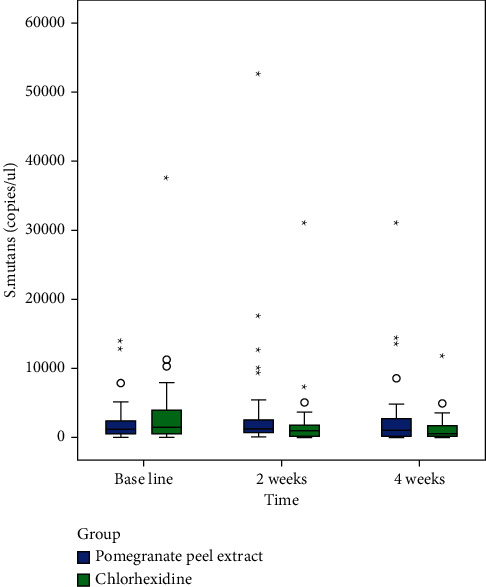
Box plot demonstrating copies of *Streptococcus mutans* at baseline, 2 weeks, and 4 weeks in both groups.

**Figure 6:**
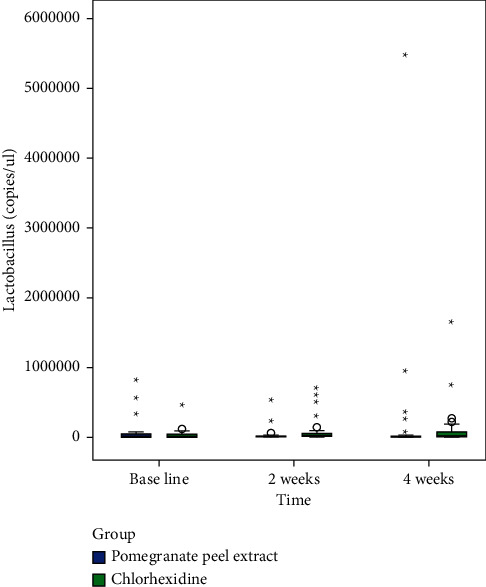
Box plot demonstrating copies of *Lactobacilli* at baseline, 2 weeks, and 4 weeks in both groups.

**Figure 7:**
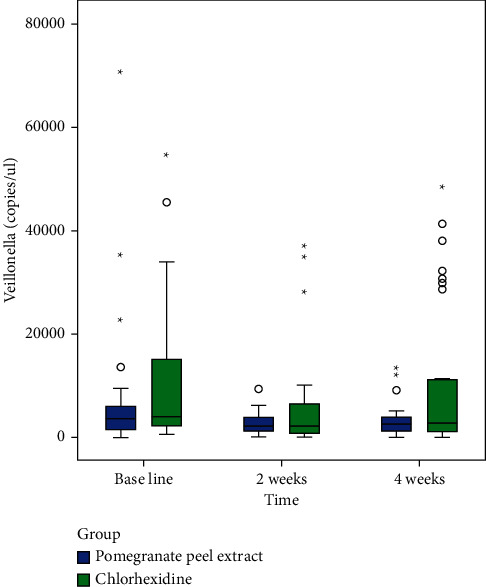
Box plot demonstrating copies of *Veillonella* at baseline, 2 weeks, and 4 weeks in both groups.

Following exclusion of 90 subjects from a total of 150 patients assessed for eligibility, random allocation of 60 subjects to a control group (*n* = 30) and an intervention group (*n* = 30) was done (shown in Figure 1). The subjects received intervention for 4 weeks, and all of them completed the experimental study. Gender variation was negligible as both groups showed equal distribution. The majority of subjects from the control group were males (56.7%), and the same was observed for females in the intervention group. There were no significant differences in age (*p*=0.916) and DMFT values (*p*=0.213) (demographic data shown in Table 2). The mean copies/*μ*l of *S. mutans*, *Lactobacilli*, and *Veillonella* were calculated at T_0_, T_1_, and T_2_. At the end of the study, on comparison between control and intervention at periodic time intervals, significant differences in the mean number of *S. mutans*, *Lactobacilli*, and *Veillonella* were not observed (shown in Table 3). Within-group analysis between variant time points (shown in Table 4) revealed a significant reduction in the mean number of *S. mutans* in samples evaluated at T_2_ in the control group following intervention (*p*=0.026); however, the same was not observed for *Lactobacilli* (*p*=0.792) and *Veillonella* (*p*=0.062). Additionally, in the control group, the results of the *Bonferroni* adjustment for pairwise comparison (shown in Table 5) revealed significant differences between baseline and 4 weeks (*p*=0.043) in the mean number of *S. mutans* when compared to baseline and 2 weeks (*p*=0.085) not to mention between 2 and 4 weeks (*p*=0.999) as well (Boxplots depicted in Figures 2–4) (Figures 5–7).

## 4. Discussion

The present study aimed to compare the antibacterial effect of chlorhexidine and Pomegranate Peel Extract oral rinse by determining the prevalence of *S. mutans*, *Lactobacilli*, and *Veillonella* in clinical salivary samples of patients with advanced stages of dental caries at baseline, two weeks and four weeks. In the present study, saliva collection was done under standardized conditions in order to maintain authenticity along with churning out reproducible results [27, 28]. A consistent period of 8–11 am was followed for the collection of saliva to minimize diurnal variations. Whole unstimulated saliva was collected from the floor of the mouth as it ensures a genuine representation of the salivary composition [29, 30]. At baseline evaluation of saliva samples, the molecular analysis revealed the prevalence of the tested microorganisms across all recruited individuals possessing high caries activity, which was also in line with earlier reported findings [31].

Following administration of control and intervention with subsequent periodic assessments, the CHX group exhibited an uninterrupted cumulative decline (62%) in *S. mutans* at T_1_ and T_2_. The PPE group saw a sharp spike in the *S. mutans* level at T_1,_ but the copies, however, plummeted (30%) when evaluated at T_2_. The above interpretation distinctly highlights the supremacy of CHX over PPE in controlling *S. mutans* levels in a short period of time. Although the antibacterial efficacy CHX against *S. mutans* activity saw a decline at T_2_, it is recommended to continue and monitor the preventive protocols for a longer period to further validate the substantive activities of both oral rinses, especially PPE. On the contrary, for *Lactobacilli*, the CHX and PPE mouthwashes responded rather unusually. Despite a rapid plunge (64%) in *Lactobacilli* copies at T_1_ of PPE administration, it was immediately followed by an abrupt escalation at T_2_. Concurrently, the CHX group displayed a slightly steady incline over the course of 1 month. Notwithstanding the fact that both groups failed to leave a lasting impact on *Lactobacilli* at the conclusion of the evaluation period, PPE oral rinse showed plausible promise compared to CHX in view of the fact that the latter did not intimidate the microbial (*Lactobacilli*) levels at any point. The samples analyzed for *Veillonella* showcased a marked slump at T_1_ for both PPE (66%) and CHX (43%) groups. Although there was an overall decline in *Veillonella* (55%) for the PPE group, the CHX group contrariwise were met with a rather brusque response at the culmination of the evaluation cycle. At T_2_, on sample analysis, the CHX group observed a soar in *Veillonella* copies which was most notably on par with the baseline level. On a comparative note, in the present study, the results demonstrated that PPE clearly outperformed CHX in curtailing *Veillonella*, highlighting the inability of CHX to immobilize the Gram-negative species.

CHX, since its inception in the 1970s, has tasted remarkable success in the dental profession, earning the eponym of the gold standard. Then again, contrasting and/or convincing evidence has been reported since the past vicennia debating the microbiocide's proximity toward the development of resistant strains of bacteria [2, 3]. Microbial resistance is a much talked about context holding significant reference to Gram-negative bacteria as opposed to the Gram-positive strains [32]. In view of the above stated, a former study confirmed the same when a dual-species biofilm composed of *S. mutans* and a strain of *Veillonella* was pitted against CHX following which *S. mutans* was intercepted but *Veillonella* rose in prominence, citing the latter's resistant temperament to the much-acclaimed antimicrobial [33]. Our investigations yielded comparable out-turns for CHX in accordance with the afore explained experiment, wherein a stark decline in *S. mutans* was out competed by a gradual but prodigious climb in *Veillonella* levels. Now, most importantly, as it is feasible for *Veillonella* to still flourish in a *Lactobacilli* dominant microbiome even at the backdrop of a waning *S. mutans* community by reason of lactic acid production and uptake, the oral microbiome will still be at risk of caries progression. *Veillonella* in company with *S. mutans* have long been contemplated as early colonizers of the oral biofilm [34, 35]. Also, several studies over the years were in acquiescence that *Lactobacillus* singly could not possibly engender dental caries but instead only thrive in a milieu already dominated by caries with MS being the driving factor for its subsequent colonization and multiplication in niche retentive sites [36]. Furthermore, in a study, also in concordance with previous investigations, it was noted that *Lactobacilli* were tenuous in colonizing and forming biofilms when alone, but in a mixed-species consortium comprising *S. mutans* and *Veillonella*, marked surges in biofilm growth were observed [37]. In a mixed-species consortium, it must be well understood that a survival strategy will come into force following the interaction of the oral biofilm with an antimicrobial [33]. The bacteria in the consortia tend to rearrange themselves spatially as a form of defense mechanism or more strikingly an adaptive response, developed to counteract the antibacterial puissance of the interventions deployed, thus expounding the resistant activity [38].

The pomegranate, scientifically known as *Punica granatum* L. of the family Punicaceae, has recently earned the cachet of being referred to as the “superfood” on account of its eternally vibrant phytochemical properties [39]. In a comparison of the total phenolic content of peel, juice, and seed from diverse pomegranate cultivars, it was demonstrated that the pomegranate peel had the highest amount of phenolic content [40]. Interestingly, phenolic compounds, which are extracted in abundance from pomegranate peels [17, 18, 41], have shown to be effective for both Gram-negative and Gram-positive species [42]. Furthermore, the antibacterial activity of PPE also depends enormously on its putative active constituents which functions to cripple not only glucan and EPS synthesis by *S. mutans* but also the growth and development of *Lactobacillus* as well [43] (described earlier). Conversely, CHX, a well-investigated microbiocide, bearing a profound affinity for *S. mutans*, is capable of binding onto the microbial cell walls, eventually creating an osmotic imbalance, and finally initiating cell demise [44, 45]. As far as substantivity is concerned, CHX is known for its inherent propensity to cohere around organic components of dentin matrix cosupported by its interaction with hydroxyapatite thereby maximizing the retentive potential in the oral cavity [46, 47]. Correspondingly, polyphenolic antioxidants or “sticky polyphenols” present in PPE oral rinse parade a natural tendency to fervently bind to oral surfaces, remaining there for extended periods [48–50]. On an interesting note, these “sticky polyphenols” undeterred by the continuous salivary rush function as “slow-release” devices ensuring polyphenol-induced antioxidant and antibacterial activity at a sedate pace [50, 51]. Polyphenols are also known to exhibit both bacteriostatic and bactericidal effects against bacteria due to their ability to chelate metal ions [42, 52]. Taken together, this probably explains the acceptable results churned out by CHX on *S. mutans* and PPE on *Lactobacilli* and *Veillonella* at varying time points.

It is important to remember that, for phytochemical analysis, alcohols are regarded as universal solvents in solvent extraction, and the selection of solvents plays a critical role in determining high extraction rates of natural plant-based products [53]. In a comparative study, it was demonstrated that the combination of ethanol and water as solvent yielded the maximum antioxidant and antibacterial activity following the extraction from pomegranate peels of the Ganesh variety [18]. Additionally, by virtue of the fact that a combination of ethanol and water results in a higher yield of polyphenols from pomegranate peel, it seems pertinent to remember that in order to release some bound phenolics, it is imperative that the extraction process is carried out using ethanol-water solvent, as ethanol improves the efficiency of extraction with water [54]. Furthermore, based on the most recent work done on zebrafish, the Organization for Economic Co-operation and Development (OECD) and European Chemicals Bureau (ECB) classified pomegranate peel ethanolic extract as safe [55]. Keeping in mind the afore discussed parameters, this study employed the Soxhlet extraction method under controlled temperatures and time (described in methodology) to obtain the HE PPE, and thereafter, the oral rinse was formulated to deliver a most distinct and patient compliant effect. Based on feedback obtained from the subjects, the CHX group reported a certain level of unpleasantness (taste), experienced during the 1^st^ week, which eventually subsided on routine usage. By contrast, in the PPE group, none of the candidates disclosed any known side effects. On intraoral examination, vivid staining of teeth was observed in a few subjects from the CHX group following the 2^nd^ follow-up period which was relatively absent in the PPE group.

In response to the cariogenic challenge, prevention and control strategies should not only focus on reversing the dysbiosis by annihilating virulent organisms but also ensure that the resident microflora is unscathed, thus assuring a healthy oral microbiome following disease eviction [56, 57]. Even though fluorides, replete with unique demineralization-remineralization potential, manifest remarkable capability in ousting cariogenic pathogens from within the oral cavity, in the wake of changing ecological percepts and contemporary dietary habits, banking on fluorides alone to the hilt may prove to be a harrowing affair like none other in times to come [58]. The past decennia have witnessed a marked transitional change in conceptualizations concerning oral disease preventive and/or control strategies eclipsing traditional overtures and centering upon natural products as the “need of the hour” [59–61]. With robust molecular advancements apropos oral disease and/or caries research in addition to a festoon of natural products analyzed to date, compelling evidence-based updates are not so far off [62, 63]. In view of the present study's empirical findings and past theoretical postulations, it has now become remotely clear that an ecological preventive strategy in conjunction with day-to-day fluoride therapy may prove to be efficacious in battling the age-old pandemic which has gripped the world over since time immemorial.

### 4.1. Future Implications

Future research (clinical trials) in caries prevention and control with natural products at the helm must also involve consortia of caries-specific microbes (apart from *S. mutans*) and perhaps an extended follow-up span as well, targeted at envisaging competitive and triumphant responses as against existing antimicrobials. Also, exploration of commensal prevalence and survival, both prior to and after oral rinse (conventional and ecological) usage, may prove to be beneficial in studying the benignant as well as detrimental microbiocide-microbiome interactions. Although we employed the conventional Soxhlet extraction process owing to its cost-effectiveness, simplicity, and high extraction efficiency, in recent years, newer and more greener methods have surfaced, overcoming significant flaws associated with their contemporaries, holding considerable promise in achieving high yields with reduced time and wastage [20, 53, 64]. It should be noted that the type of pomegranate cultivar selected, solvents chosen, and variant modes of extraction employed, not to mention the concentration of extract used for mouthwash formulation, can produce a legion of outcomes (antibacterial effects). Further studies can and should focus upon comparing conventional and other techniques in the extraction of essential compounds from pomegranate fruits of variant cultivars and emphasize the final concentration used as well, to standardize the selection process thereby generating significant and less confounding ramifications.

## 5. Conclusion

In conclusion, the overall comparison of antimicrobial effectiveness across both groups revealed the absence of evident changes; however, as far as an oral rinses' ability to enforce a more sustained effect on *S. mutans* is concerned, according to the results produced in this study, CHX on that aspect was still shown to be ahead of the curve. PPE oral rinse as a natural product or ecological alternative was effective in disrupting activity across all microorganisms (*Veillonella* and *Lactobacilli* in particular) tested in this triple-blind RCT at most points in time; however, the nutraceutical, when compared to chlorhexidine, was not as effective against *S. mutans*.

## Figures and Tables

**Table 1:** Primer sequences used to analyze the quantitative presence in the saliva of patients.

Bacteria	Primer sequence
*S. mutans*	GGTCAGGAAAGTCTGGAGTAAAAGGCTA
	GCGTTAGCTCCGGCACTAAGCC

*Veillonella*	CCGTGATGGGATGGAAACTGC
	CCTTCGCCACTGGTGTTCTTC

*Lactobacillus*	AGCAGTAGGGAATCTTCCA
	CACCGCTACACATGGAG

**Table 2:** Demographic characteristics of the subjects.

Variable	Intervention group (*n* = 30)	Control group (*n* = 30)	*p* value
Age (years)	37.40 ± 9.995	37.13 ± 9.562	0.916
Male (number)	13	17	0.302
Female (number)	17	13	
DMFT	10.1 ± 2.5	9.2 ± 1.7	0.213

**Table 3:** Mann–Whitney test to compare *S. mutans*, *Lactobacilli*, and *Veillonella* values between groups.

		Group		*p* value
		PPE	CHX	
*S. mutans* (copies/*μ*l): baseline	*N*	30	30	0.455
	Mean	2708.0	3772.2	
	Std. Dev	4028.5	7032.0	

*S. mutans* (copies/*μ*l): 2 weeks	*N*	30	30	0.169
	Mean	4557.0	2367.0	
	Std. Dev	9983.4	5660.2	

*S. mutans* (copies/*μ*l): 4 weeks	*N*	30	30	0.483
	Mean	3216.9	1414.1	
	Std. Dev	6354.6	2310.1	

*Lactobacillus* (copies/*μ*l): baseline	*N*	30	30	0.756
	Mean	82149.5	43010.3	
	Std. Dev	188267.5	90490.3	

*Lactobacillus* (copies/*μ*l): 2 weeks	*N*	30	30	0.064
	Mean	29718.9	89112.6	
	Std. Dev	95025.8	179892.1	

*Lactobacillus* (copies/*μ*l): 4 weeks	*N*	30	30	0.460
	Mean	244541.2	124800.4	
	Std. Dev	1007671.1	323234.8	

*Veillonella* (copies/*μ*l): baseline	*N*	30	30	0.344
	Mean	7691.5	10268.8	
	Std. Dev	13925.2	13609.0	

*Veillonella* (copies/*μ*l): 2 weeks	*N*	30	30	0.767
	Mean	2643.5	5861.1	
	Std. Dev	2249.5	9794.6	

*Veillonella* (copies/*μ*l): 4 weeks	*N*	30	30	0.469
	Mean	3471.4	10494.6	
	Std. Dev	3630.5	14735.4	

**Table 4:** The Friedman test to compare *S. mutans*, *Lactobacilli*, and *Veillonella* values between time points.

		Group		*p* value
		PPE	CHX	
*S. mutans* (copies/*μ*l): baseline	*N*	30	30	PPE
	Mean	2708.0	3772.2	0.741
	Std. Dev	4028.5	7032.0	
*S. mutans* (copies/*μ*l): 2 weeks	*N*	30	30
	Mean	4557.0	2367.0
	Std. Dev	9983.4	5660.2	CHX
*S. mutans* (copies/*μ*l): 4 weeks	*N*	30	30	0.026
	Mean	3216.9	1414.1	
	Std. Dev	6354.6	2310.1	

*Lactobacillus* (copies/*μ*l): baseline	*N*	30	30	PPE
	Mean	82149.5	43010.3	0.291
	Std. Dev	188267.5	90490.3	
*Lactobacillus* (copies/*μ*l): 2 weeks	*N*	30	30
	Mean	29718.9	89112.6
	Std. Dev	95025.8	179892.1	CHX
*Lactobacillus* (copies/*μ*l): 4 weeks	*N*	30	30	0.792
	Mean	244541.2	124800.4	
	Std. Dev	1007671.1	323234.8	

*Veillonella* (copies/*μ*l): baseline	*N*	30	30	PPE
	Mean	7691.5	10268.8	0.497
	Std. Dev	13925.2	13609.0	
*Veillonella* (copies/*μ*l): 2 weeks	*N*	30	30
	Mean	2643.5	5861.1
	Std. Dev	2249.5	9794.6	CHX
*Veillonella* (copies/*μ*l): 4 weeks	*N*	30	30	0.062
	Mean	3471.4	10494.6	
	Std. Dev	3630.5	14735.4	

**Table 5:** Bonferroni adjusted Wilcoxon signed rank test to compare pairwise time points.

Group	Time points	*p* value
Chlorhexidine	Baseline vs. 2 weeks	0.085
	Baseline vs. 4 weeks	0.043
	2 weeks vs. 4 weeks	0.999

## Data Availability

Data are available on request.
